# Evaluation of Mortality and Hospitalization Due to Decompensated Heart Failure and Appropriate Shocks in Reduced Ejection Fraction in Patients with an Implantable Cardioverter–Defibrillator According to a Novel Tissue Doppler Echocardiographic Method

**DOI:** 10.3390/jcm14093226

**Published:** 2025-05-06

**Authors:** Gökhun Akkan, Tuncay Kiris, Fatma Esin, Mustafa Karaca

**Affiliations:** 1 Department of Cardiology, Nazilli State Hospital, Aydın 09800, Turkey; gokhunakkan54@gmail.com; 2 Department of Cardiology, Atatürk Training and Research Hospital, Izmir Katip Çelebi University, Izmir 35360, Turkey; drfatmakesin@gmail.com (F.E.); mustafakaraca99@hotmail.com (M.K.)

**Keywords:** implantable cardioverter–defibrillator, pulmonary capillary wedge pressure, transthoracic echocardiography, IVCDi × ST/SM, mortality, heart failure

## Abstract

**Background/Objectives:** Heart failure is a very common disease, and its incidence is increasing. Echocardiography is a non-invasive tool frequently used in the diagnosis and risk stratification of heart failure. In our study, we aimed to evaluate the risk of all-cause mortality, hospitalization due to decompensated heart failure, and appropriate shocks in reduced ejection fraction patients (HFrEF) with an implantable cardioverter–defibrillator (ICD) according to a novel tissue Doppler echocardiographic parameter that reflects pulmonary capillary wedge pressure. **Methods**: A total of 320 HFrEF patients with ICD were included in the study between 1 February 2021 and 30 June 2023, from the cardiology outpatient clinic and cardiology ward. Using tissue Doppler, the peak systolic velocity (ST) at the free wall side of the tricuspid annulus and the peak systolic velocity (SM) at the lateral side of the mitral annulus were measured, and the ratio of ST to SM (ST/SM) was calculated. The inferior vena cava diameter (IVCDi) was measured during inspiration. These two values were multiplied to form the formula IVCDi × (ST/SM). Based on the IVCDi × (ST/SM) value, patients were divided into two groups: those with high values (>17, *n* = 144) and those with low values (≤17, *n* = 176). The primary endpoint of our study was all-cause mortality, and the secondary endpoint was major adverse cardiovascular events (MACE), including appropriate shocks, hospital admission due to acute heart failure decompensation, and mortality. **Results**: Long-term mortality was higher in the high IVCDi × (ST/SM) group compared to the low-value group (44% vs. 15%, *p* < 0.001). The MACE frequency was also higher in patients with high IVCDi × (ST/SM) values (71% vs. 30%, *p* < 0.001). In multivariable analysis, IVCDi × (ST/SM) was an independent predictor of both mortality (HR: 1.027, 95%CI: 1.009–1.046, *p* = 0.003), and MACE (HR: 1.018, 95%CI: 1.004–1.032, *p* = 0.013). **Conclusions**: We demonstrated that the IVCDi × ST/SM value, a novel tissue Doppler echocardiographic parameter, is an independent predictor of both long-term mortality and major adverse cardiac events (MACE) in HFrEF patients with ICD. This parameter may be valuable in identifying high-risk patients and optimizing their treatment management.

## 1. Introduction

The incidence of heart failure (HF) in Europe is approximately 3 per 1000 person-years across all age groups and 5 per 1000 person-years in adults [1]. The prevalence of HF is estimated at 12% of adults [2], though the true rate may be higher since studies typically include only diagnosed cases [3]. A combined analysis of the Framingham Heart Study and Cardiovascular Health Study reported a 67% mortality rate within five years of HF diagnosis [4].

Pulmonary capillary wedge pressure (PCWP) reflects left atrial pressure and left ventricular end-diastolic pressure [5]. Several echocardiographic methods have been developed for noninvasive estimation of PCWP [6,7,8,9,10,11]. Among them, the ratio of early transmitral velocity to tissue Doppler mitral annular early diastolic velocity (E/Ea) is one of the most known and used methods [9,10,11]. Nevertheless, the reliability of these echocardiographic methods remains a major issue [12,13].

Chinen and colleagues developed a novel echocardiographic method to calculate pulmonary capillary wedge pressure (PCWP) [14]. According to this model, the inspiratory inferior vena cava diameter (IVCDi) is measured and multiplied by the ratio of peak systolic velocity of the tricuspid annulus (ST) to that of the mitral annulus (SM). This method has demonstrated high predictive value in estimating PCWP, particularly in patients with heart failure and reduced ejection fraction (HFrEF) [14]. Their study demonstrated that an IVCDi × (ST/SM) value > 17 was positively correlated with elevated PCWP (>18 mmHg) in HFrEF patients.

The aim of this study was to evaluate the role of the novel echocardiographic method in predicting mortality, hospitalization due to decompensated heart failure, and appropriate shocks in long-term follow-up in HFrEF patients with an implantable cardioverter–defibrillator (ICD).

## 2. Materials and Methods

### 2.1. Study Patients, and Design

We enrolled 320 patients (70 patients were included in the study retrospectively, and 250 patients were included prospectively) aged 18 years or older admitted to our institution with an ICD and a diagnosis of HFrEF between 1 February 2021 and 30 June 2023. Exclusion criteria were as follows: patients with left ventricular ejection fraction (LVEF) > 35% (*n* = 18), severe valvular diseases (*n* = 7), prosthetic valves (*n* = 4), acute coronary syndrome (*n* = 5), atrial septal defect (*n* = 2), coronary to pulmonary artery fistula (*n* = 1), patients whose data could not be accessed during follow-up (*n* = 3).

### 2.2. Echocardiography

With the patient in a steady state, echocardiography was performed using the GE Healthcare Vivid E95 (Horten, Norway) cardiovascular ultrasound system. Two-dimensional images were acquired in supine position using a phased array transducer in the standard parasternal, apical, and subcostal views. From the subcostal view, longitudinal image of the inferior vena cava was recorded throughout the respiratory cycle. From the apical 4-chamber view, the pulsed Doppler sample volume was placed at the mitral valve tip, and mitral inflow was recorded. From the apical 4-chamber view, the tricuspid and mitral annular velocities were obtained with pulsed tissue Doppler by placing 5 mm wide sample volumes at the free wall side of the tricuspid annulus and at the medial and lateral sides of the mitral annulus, respectively. Gains were adjusted to eliminate background noise and obtain clear tissue signals. A range of 5 to 10 cardiac cycles was recorded. Intraobserver and interobserver variabilities were evaluated in 12 randomly selected patients. Intraobserver variability was assessed by repeating the measurements from video recordings at two different time points, two weeks apart. To assess interobserver variability, the measurements were performed from video recordings by a second observer who was unaware of the results of the initial examination. All reported echocardiographic data were averaged from 3 consecutive cycles in sinus rhythm and 7 consecutive cycles in atrial fibrillation. The LV dimension, volume, ejection fraction, and severity of valvular disease were assessed in accordance with the American Society Echocardiography Guidelines [15,16]. The IVCD at inspiration (IVCDi) and at expiration (IVCDe) were measured within 2 cm from the IVC-right atrial junction. Peak velocity of early (E) filling was calculated. The following measurements were obtained from the tissue Doppler: peak systolic velocities at free wall side of the tricuspid annulus (ST) and at lateral side of the mitral annulus (SM) and early (Ea) diastolic velocities at medial and lateral sides of the mitral annulus. Data of Ea represent the average of Ea measured at the medial and lateral mitral annulus.

### 2.3. Data Collection

Data collected included the patients’ demographics (age, gender, and history), the type of ICD implanted (VVI, DDD, or cardiac resynchronization therapy [CRT]), and the etiology of their heart failure (ischemic or non-ischemic). In addition, patients were categorized according to New York Heart Association (NYHA) classes, their electrocardiography (EKG) rhythms were documented, and details of their medical treatments were recorded. Measurements such as brain natriuretic peptide (BNP) levels, left ventricular ejection fraction (LVEF), tricuspid annular plane systolic excursion (TAPSE), Ea, E-wave velocity, and systolic pulmonary artery pressures (SPAP) (as determined by echocardiography) were obtained, along with IVCDi × (ST/SM) values measured using the previously described echocardiographic method.

The patients were divided into 2 groups based on IVCDi × (ST/SM) values: IVCDi × (ST/SM) > 17 (high-value group, *n* = 144) and IVCDi × (ST/SM) ≤ 17 (low-value group, *n* = 176). The patients were followed up and recorded for appropriate shocks, emergency department admissions due to decompensation, and mortality.

### 2.4. Study Endpoints

The primary endpoint of our study was all-cause mortality, and the secondary endpoint was major adverse cardiovascular events (MACE), including appropriate shocks, hospital admission due to acute heart failure decompensation, and mortality, whichever occurred first. The last follow-up was set for 1 December 2023.

### 2.5. Statistical Analysis

Descriptive statistics were presented as mean and standard deviation for continuous variables and as counts and percentages for categorical variables. The normal distribution of the data was assessed using the Kolmogorov–Smirnov test. Variables following a normal distribution were expressed as mean ± SD, while those not following a normal distribution were presented as median (min-max). For comparisons between groups, the Pearson Chi-Square test was used for categorical variables, while Student’s *T*-test and Mann–Whitney U test were used for numerical variables. Correlation analysis between IVCDi × (ST/SM) and other parameters was carried out by Pearson or Spearman test. Univariate and multivariate Cox regression analyses were performed to search for predictors of long-term mortality and MACE. Intraobserver variability was assessed using the intraclass correlation coefficient (ICC). ICC values below 0.5 indicated poor reliability, those between 0.5 and 0.75 indicated moderate reliability, those between 0.75 and 0.9 indicated good reliability, and those exceeding 0.90 indicated excellent reliability [17]. The kappa statistic was used to test interobserver variability. The kappa values were interpreted as follows: <0.20 poor or slight; 0.21–0.40 weak; 0.41–0.60 moderate; 0.61–0.80 good; 0.81–1 almost perfect agreement [18]. A *p*-value of < 0.05 was considered statistically significant for all statistical analyses. All statistical analyses were conducted using SPSS software, version 26 (SPSS Inc., Chicago, IL, USA), and R software version 4.1.2.

## 3. Results

### 3.1. Patients Characteristics

Median follow-up time was 15.6 (7.3–21.7) months. Follow-up data were available for all patients included in the study. The clinical characteristics of the 320 patients are shown in Table 1. The number of patients with NYHA ≥ III was higher in the high-value group (39% vs. 15%, *p* < 0.001). No difference was observed between the groups in terms of ACEi/ARB usage (64% vs. 72%, *p* = 0.141). When compared in terms of the usage of loop diuretics, their usage was higher in the high-value group (83% vs. 71%, *p* = 0.011). The laboratory and echocardiographic findings of the patients are presented in Table 2. At the time the patients were enrolled in the study, BNP values were higher in the high-value group (1378 [538–3380] vs. 375 [144.5–833.5], *p* < 0.001). In the high-value group, patients had lower LVEF values (24.3 ± 5.3% vs. 27.6 ± 5.0%, *p* < 0.001).

Table 3 presents the correlations between the IVCDi × (ST/SM) value and other parameters. The analysis revealed that the IVCDi × (ST/SM) value had positive correlations with SPAB, BNP, NYHA class, E-wave velocity, and E/Ea (r = 0.342, r = 0.464, r = 0.304, r = 0.241, and r = 0.362, respectively; all *p* < 0.001). In contrast, significant negative correlations were observed with LVEF, TAPSE, and Ea velocity (r = −0.304, r = −0.191, and r = −0.204, respectively; all *p* < 0.001).

**Figure 1:**
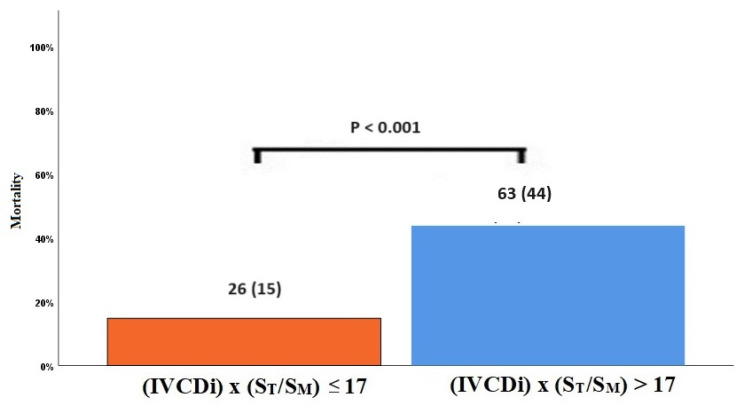
Mortality rates in high and low IVCDi × (ST/SM) patients.

**Figure 2:**
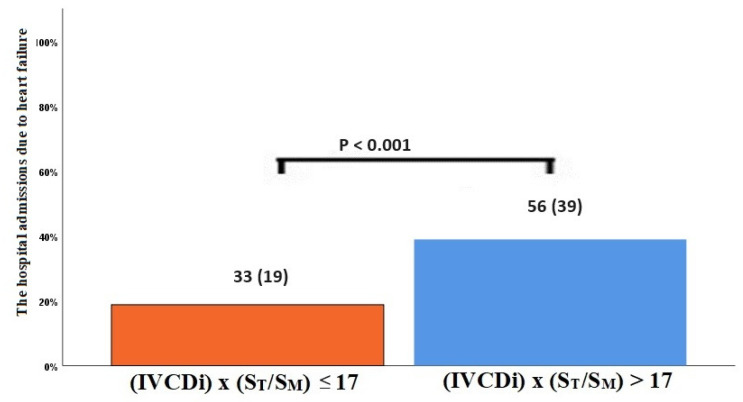
Hospitalization due to decompensated heart failure according to IVCDi × (ST/SM) patients.

**Figure 3:**
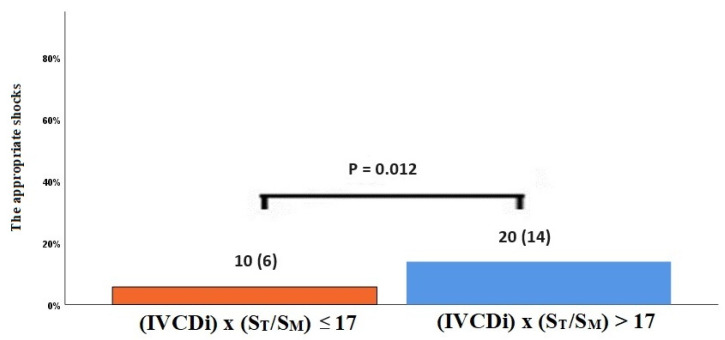
The appropriate shock rates in high and low IVCDi × (ST/SM) patients.

**Figure 4:**
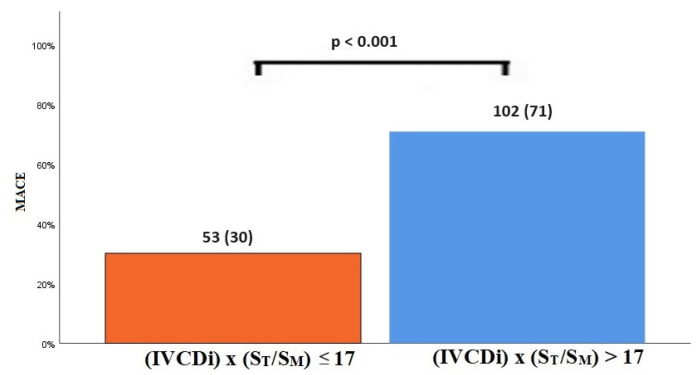
MACE in high and low IVCDi × (ST/SM) patients.

The data regarding primary and secondary endpoints are shown in Table 4 and Figure 1, Figure 2, Figure 3 and Figure 4. Long-term mortality was higher in the high-value group compared to the low-value group (44% vs. 15%, *p* < 0.001, Figure 1). The frequency of hospital admissions due to heart failure was observed to be higher in the high-value group (39% vs. 19%, *p* < 0.001, Figure 2). In the high-value group, the appropriate shocks were observed more frequently (14% vs. 6%, *p* = 0.012, Figure 3). The frequency of MACE was higher in the high-value group (71% vs. 30%, *p* < 0.001, Figure 4).

### 3.2. Prognostic Impact of IVCDi × ST/SM in HFrEF Patients with ICD

In multivariate analysis, IVCDi × ST/SM was an independent predictor of mortality (HR: 1.027, 95%CI: 1.009–1.046, *p* = 0.003, Table 5). Also, in the multivariate analysis where all echo parameters were taken together, IVCDi × ST/SM was associated with long-term mortality (HR:1.034, 95%CI: 1.06–1.053, *p* < 0.001, Table 6). Similarly, IVCDi × ST/SM was independently and positively correlated with the MACE (HR: 1.018, 95%CI: 1.004–1.032, *p* = 0.013, Table 7). Moreover, we found that IVCDi × ST/SM was independently related to MACE in the analysis made with all echo parameters (HR:1.017, 95%CI:1.003–1.032, *p* = 0.021, Table 8).

**Figure 5:**
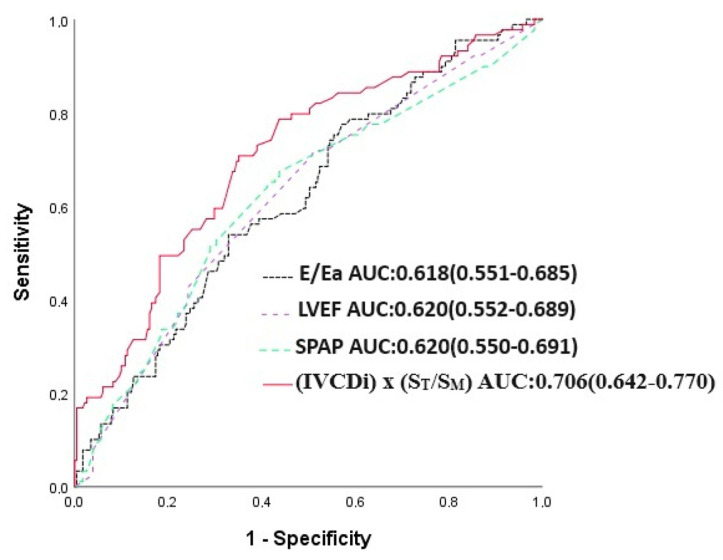
E/Ea, LVEF, SPAP, and IVCDi ×ST/SM receiver operating characteristic (ROC) in predicting mortality.

**Figure 6:**
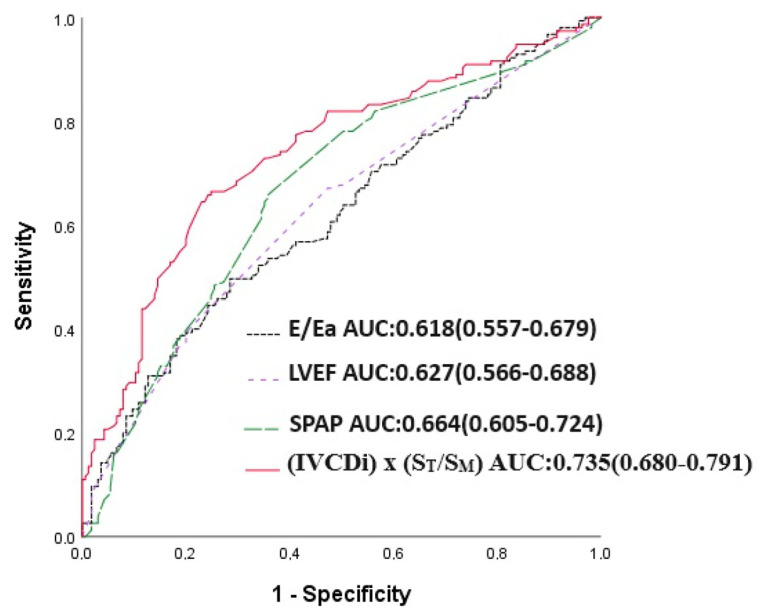
E/Ea, LVEF, SPAP, and IVCDi ×ST/SM receiver operating characteristic (ROC) in predicting MACE.

The IVCDi × ST/SM parameter demonstrated a stronger predictive value for mortality compared to LVEF, E/Ea, and SPAB. The area under the curve (AUC) for IVCDi × ST/SM was 0.706, significantly higher than 0.618 for E/Ea (*p* = 0.023), 0.620 for SPAB (*p* = 0.043), and 0.620 for LVEF (*p* = 0.036) (Figure 5). Similarly, IVCDi × ST/SM showed superior predictive performance for major adverse cardiac events (MACE), with an AUC of 0.735, compared to 0.618 for E/Ea (z = 3.266, difference *p* = 0.001), 0.664 for SPAB (z = 1.978, difference *p* = 0.048), and 0.627 for LVEF (z = 1.988, difference *p* = 0.003) (Figure 6).

### 3.3. Survival Analysis

**Figure 7:**
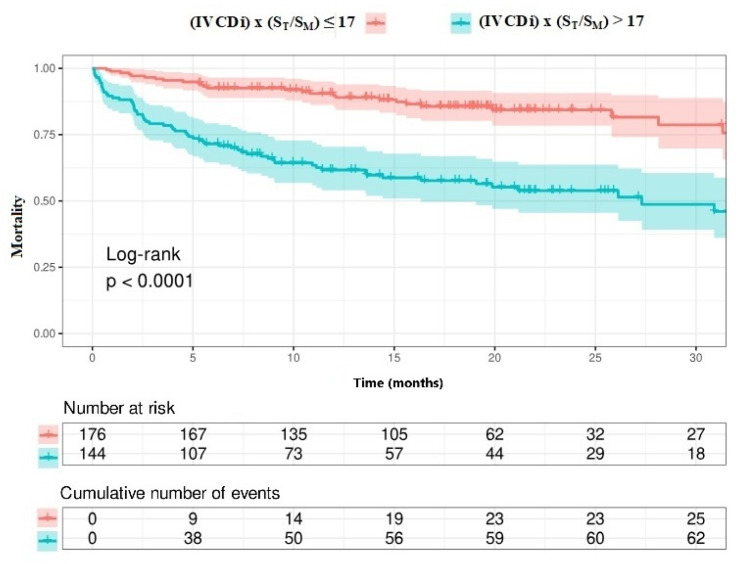
Survival analysis according to IVCDi × S_T_/S_M_ value.

**Figure 8:**
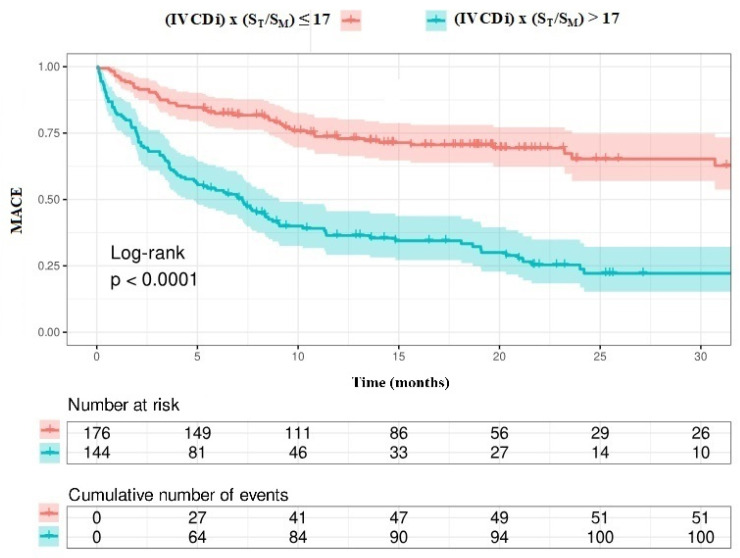
MACE analysis according to IVCDi ×S_T_/S_M_ value.

Patients with high IVCDi × ST/SM values had a decreased long-term life expectancy (Figure 7). Additionally, patients with high IVCDi × ST/SM values had a higher frequency of cardiovascular events in terms of MACE (Figure 8).

### 3.4. Evaluation of Interobserver Agreement

The intraclass correlation coefficient was 0.932 (95%CI:0725–0.981, *p* < 0.001) for ST, 0.934 (95%CI:0.782–0.981, *p* < 0.001) for SM, 0.997 (95%CI:0.988–0.999, *p* < 0.001) for IVCDi, and 0.940 (95%CI:0.780–0.983, *p* < 0.001) for IVCDi × (ST/SM).

Kappa statistics for interobserver agreement was 0.808 (standard error: 0.120, *p* < 0.001) for ST, 0.788 (standard error: 0.130, *p* < 0.001) for SM, 0.814 (standard error: 0.116, *p* < 0.001) for IVCDi, and 0.647 (standard error: 0.136, *p* < 0.001) for IVCDi × (ST/SM) suggesting good interobserver agreement.

## 4. Discussion

According to our best knowledge, this is the first study to investigate the relationship between IVCDi × ST/SM as a new echocardiographic parameter and long-term outcomes in patients with HFrEF. In this study, we demonstrated that IVCDi × ST/SM, shown to be most strongly correlated with PCWP, was an independent predictor of both mortality and MACE in these patients during long-term follow-up. IVCDi × ST/SM was superior to other echocardiographic parameters such as LVEF, E/Ea, and SPAB in predicting long-term outcomes.

PCWP is a hemodynamic parameter that reflects the compliance of the left side of the heart and is closely associated with left atrial pressure. This parameter is clinically used to assess left ventricular filling pressures and to analyze the impact of left heart function on pulmonary circulation. Therefore, it is a parameter that can assist physicians in diagnosis and treatment. Pulmonary congestion often develops due to an increase in PCWP in acute decompensated heart failure [19]. Among patients hospitalized due to heart failure, persistent high PCWP and residual pulmonary congestion at discharge are strongly associated with high mortality and readmission rates [19]. Therefore, reducing congestion in patients hospitalized for acute decompensated heart failure (ADHF) is one of the most important therapeutic goals, and it is important to reliably detect and monitor pulmonary congestion before discharge in these patients [20]. However, quantitative assessment of pulmonary congestion is often challenging, and it is reported that approximately half of patients presenting with ADHF are discharged with residual congestive disease [21]. The gold standard method for measuring PCWP is through right heart catheterization via pulmonary artery catheterization. However, the placement and management of pulmonary artery catheters can be challenging due to complications. Chest radiography is a rapid, simple, and classic method for assessing elevated PCWP and pulmonary congestion in heart failure [22]; however, the interpretation of chest radiographs is subjective and may not always ensure an accurate assessment of PCWP. For this reason, alternative non-invasive methods have become more widely used. Echocardiographic E/Ea has started to be used as a reliable indicator of PCWP. Measurement of E/Ea is one of the methods recommended by the 2016 ASE/EACVI guidelines for the assessment of left ventricular diastolic dysfunction [23]. In a meta-analysis conducted by Jones and colleagues, comprising 17 studies and 1348 patients, a significant correlation was demonstrated between E/Ea and invasively measured PCWP [24]. Another meta-analysis by Nauta and colleagues, including nine studies, showed a positive moderate-level correlation between E/Ea and PCWP in heart failure patients [25].

In a study by Chien and colleagues involving 98 patients diagnosed with heart failure, a newly introduced echocardiographic parameter was correlated with invasively measured PCWP [14]. In that study, they demonstrated that the IVCDi × ST/SM value obtained using tissue Doppler had a strong correlation with PCWP. Additionally, this parameter outperformed E/Ea in predicting PCWP. This model was particularly beneficial in patients with LVEF ≤ 35%. In their study, specifically, the IVCDi × ST/SM = 17 value had high sensitivity and specificity for elevated PCWP.

In our study, patients with high IVCDi × ST/SM values had a higher mortality rate and were more likely to experience the composite endpoint consisting of hospitalization due to decompensated heart failure, VT shock, and total mortality. In addition to having elevated pulmonary capillary wedge pressure, these patients also showed correlations between IVCDi × ST/SM values and several parameters previously linked to poor cardiovascular outcomes, including chronic kidney disease (CKD), NYHA class, BNP levels, increased SPAP, and reduced LVEF. The presence of these variables may contribute to the increased risk of adverse cardiovascular events in this patient group.

In recent years, numerous studies have been published comparing modern drug therapies in patients with HFrEF [26,27]. A recent meta-analysis examining data from trials such as PARADIGM-HF [28], DAPA-HF [29], EMPEROR reduced [30], VICTORIA [31], and GALACTIC-HF [32] has provided insights into which medications are effective, particularly in specific subgroups of patients (e.g., the elderly, patients with CKD, DM, CAD, NYHA class III/IV, women) [33]. In that study, especially the use of sodium-glucose cotransporter 2 inhibitors (SGLT-2) and angiotensin receptor-neprilysin inhibitors (ARNI) in these patients has been associated with improved cardiovascular outcomes [33].

Our study has some limitations. It is single-center and relatively small in sample size. All four foundational pillars of pharmacologic guideline-directed medical therapy (GDMT), including ACE-I/ARB/angiotensin receptor-neprilysin inhibitors (ARNIs), β-blockers (BBs), mineralocorticoid receptor antagonists (MRA), and SGLT2- inhibitors have demonstrated a significant reduction in morbidity and mortality. Concurrent use of all four drug classes has been estimated to reduce all-cause mortality by 73% in patients with HFrEF [34]. The utilization rate of SGLT-2 inhibitors and ARNI was very low in our study. Therefore, in our patient population, the markedly low usage of these medications, which have demonstrated beneficial effects, particularly on cardiovascular outcomes, limits the generalizability of this study’s findings. The exclusion of patients with severe valve disease, prosthetic valves, and acute coronary syndrome makes it difficult to provide information regarding the use of this parameter in these patients. Since we lack data on right heart catheterization in these patients, it is difficult to make a definitive interpretation regarding the level of correlation between IVCDi × ST/SM and PCWP. Large, multicenter studies are needed to clearly establish the relationship between IVCDi × ST/SM and long-term cardiovascular outcomes.

## 5. Conclusions

In our study, we demonstrated that in patients with HFrEF and those with ICDs, the newly introduced echocardiographic parameter IVCDi × ST/SM had independent predictive value for both primary and secondary endpoints. In this patient subgroup, IVCDi × ST/SM outperformed commonly used echocardiographic parameters such as E/Ea, SPAB, and LVEF in predicting endpoints.

## Figures and Tables

**Table 1:** Baseline characteristics of the study population.

Variables	(IVCDi) × (S_T_/S_M_) ≤ 17 (*n* = 176)	(IVCDi) × (S_T_/S_M_) > 17 (*n* = 144)	*p*-Value
Age, years	62.3 ± 10.5	62.5 ± 12.0	0.911
Female gender (%)	28 (16)	20 (14)	0.615
Hypertension, *n* (%)	94 (53)	73 (51)	0.629
Diabetes mellitus, *n* (%).	66 (38)	61 (42)	0.377
Previous CKD, *n* (%)	25 (14)	44 (31)	<0.001
Atrial fibrillation, *n* (%)	24 (14)	42 (29)	0.001
Asthma or COPD, *n* (%)	27 (15)	15 (10)	0.194
Previous CAD, *n* (%)	129 (73)	99 (69)	0.371
Previous stroke/TIA, *n* (%)	18 (10)	15 (10)	0.956
NYHA ≥ III, *n* (%)	26 (15)	56 (39)	<0.001
Ischemic cardiomyopathy, *n* (%)	128 (73)	99 (69)	0.428
CRT, *n* (%)	30 (17)	21 (15)	0.549
Medical treatment, *n* (%)			
B-blockers	169 (96)	133 (92)	0.157
ACEi/ARB	126 (72)	92 (64)	0.141
MRA	132 (75)	89 (62)	0.011
Loop diüretics	124 (71)	119 (83)	0.011
Digitalis	6 (3)	13 (9)	0.034
İvabradine	26 (15)	23 (16)	0.767
Amiodarone	22 (13)	39 (28)	0.001
SGLT-2 inhibitors	30 (17)	28 (19)	0.579
ARNI	9 (5)	11 (8)	0.353

Abbreviations: ACE-I/ARB; angiotensin-converting enzyme inhibitors/angiotensin receptor blocker, AF; atrial fibrillation, ARNI; angiotensin receptor–neprilysin inhibitor, CAD; coronary artery disease, CKD; chronic kidney disease, COPD; chronic obstructive pulmonary disease, CRT; cardiac resynchronization therapy, NHYA; New York heart association, LVEF; left ventricular ejection fraction, MRA; mineralocorticoid receptor antagonists, SGLT; sodium-glucose cotransporter, TIA; transient ischemic attack, IVCDi; inspiratory inferior vena cava diameter, S_M_; mitral annulus peak systolic velocity, S_T_; tricuspid annulus peak systolic velocity.

**Table 2:** Laboratory and echocardiographic findings of patients.

Variables	(IVCDi) × (S_T_/S_M_) ≤ 17 (*n* = 176)	(IVCDi) × (S_T_/S_M_) > 17 (*n* = 144)	*p*-Value
BNP (ng/L)	375 (144.5–833.5)	1378 (538–3380)	<0.001
LVEF (%)	27.6 ± 5.0	24.3 ± 5.3	<0.001
E velocity, cm/s	85.0 ± 27.3	97.0 ± 23.3	<0.001
Ea velocity, cm/s	7.9 ± 2.5	6.7 ± 2.3	<0.001
E/Ea	11.6 ± 4.7	16.0 ± 6.5	<0.001
S_M_, cm/s	5.2 ± 1.4	4.6 ± 1.3	<0.001
S_T_, cm/s	9.4 ± 2.3	9.5 ± 3.0	0.688
S_T_/S_M_	1.9 ± 0.5	2.1 ± 0.7	<0.001
TAPSE, mm	16.7 ± 3.3	15.0 ± 4.2	0.001
IVCDi, mm	6.6 ± 2.1	14.5 ± 6.0	<0.001
SPAB (mm Hg)	31.7 ± 11.4	39.3 ± 11.9	<0.001
IVCDi × S_T_/S_M_, mm	11.8 ± 3.3	28.8 ± 11.2	<0.001

Abbreviations: BNP; brain natriuretic peptide, E; early diastolic transmitral wave velocity, Ea; tissue Doppler mitral annular early diastolic velocity, LVEF; left ventricular ejection fraction, S_M_; mitral annulus peak systolic velocity, SPAB; systolic pulmonary artery pressure, S_T_; tricuspid annulus peak systolic velocity, TAPSE; tricuspid annular plane systolic excursion, IVCDi; inspiratory inferior vena cava diameter.

**Table 3:** Correlations of (IVCDi) × (S_T_/S_M_) with other variables.

Variables	r-Value	*p*-Value
SPAB (mm Hg)	0.342	<0.001
E velocity, cm/s	0.241	<0.001
Ea velocity, cm/s	−0.204	<0.001
E/Ea	0.362	<0.001
LVEF(%)	−0.304	<0.001
TAPSE	−0.191	<0.001
BNP (ng/L)	0.464	<0.001
NYHA sınıfı	0.304	<0.001

Abbreviations: BNP; brain natriuretic peptide, E; early diastolic transmitral wave velocity, Ea; tissue Doppler mitral annular early diastolic velocity, LVEF; left ventricular ejection fraction, NYHA; New York heart association, S_M_; mitral annulus peak systolic velocity, SPAB; systolic pulmonary artery pressure, S_T_; tricuspid annulus peak systolic velocity, TAPSE; tricuspid annular plane systolic excursion, IVCDi; inspiratory inferior vena cava diameter.

**Table 4:** Long-term outcomes.

Outcomes	(IVCDi) × (S_T_/S_M_) ≤ 17 (*n* = 176)	(IVCDi) × (S_T_/S_M_) > 17 (*n* = 144)	*p* Value
MACE, *n* (%)	53 (30)	102 (71)	<0.001
Heart failure decompensation, *n* (%)	33 (19)	56 (39)	<0.001
Appropriate shock occurrence, *n* (%)	10 (6)	20 (14)	0.012
Long-term mortality, *n* (%)	26 (15)	63 (44)	<0.001

Abbreviations: MACE; major cardiovascular events, S_M_; mitral annulus peak systolic velocity, S_T_; tricuspid annulus peak systolic velocity, IVCDi; inspiratory inferior vena cava diameter.

**Table 5:** Cox proportional hazards models to identify independent predictors of all-cause mortality.

Univariate						Multivariate				
			Model 1		Model 2		Model 3		Model 4	
Variables	HR (95%CI)	*p*	HR (95%CI)	*p*	HR (95%CI)	*p*	HR (95%CI)	*p*	HR (95%CI)	*p*
Age (years)	1.009 (1.030–1.052)	0.005	1.012 (0.990–1.034)	0.284	1.007 (0.986–1.029)	0.526	1.007 (0.986–1.030)	0.508	1.012 (0.989–1.035)	0.313
Previous CKD	3.492 (2.284–5.341)	<0.001	1.955 (1.111–3.439)	0.020	1.720 (0.989–2.991)	0.055	1.731 (1.003–2.989)	0.049	1.953 (1.118–3.412)	0.019
NYHA class	3.314 (2.397–4.581)	<0.001	2.364 (1.647–3.392)	<0.001	2.580 (1.791.3715)	<0.001	2.529 (1.777–3.599)	<0.001	2.248 (1.553–3.245)	<0.001
ACE-I/ARB usage	0.404 (0.266–0.615)	<0.001	0.622 (0.387–1.001)	0.050	0.607 (0.369–0.996)	0.048	0.629 (0.388–1.017)	0.059	0.644 (0.402–1.032)	0.067
Loop diuretıcs usage	2.735 (1.452–5.152)	0.002	1.679 (0.833–3.384)	0.147	1.642 (0.809–3.332)	0.170	1.677 (0.831–3.383)	0.149	1.627 (0.799–3.315)	0.180
MRA usage	0.664 (0.434–1.017)	0.060	1.283 (0.754–2.182)	0.359	1.231 (0.728–2.081)	0.438	1.292 (0.763–2.187)	0.341	1.437 (0.837–2.468)	0.189
BNP	1.000 (1.000–1.000)	<0.001	1.000 (1.000–1.000)	0.001	1.000 (1.000–1.000)	<0.001	1.000 (1.000–1.000)	<0.001	1.000 (1.000–1.000)	0.003
Appropriate shock	1.783 (1.005–3.160)	0.048	2.236 (1.212–4.125)	0.010	2.116 (1.150–3.894)	0.016	2.084 (1.127–3.856)	0.019	1.901 (1.027–3.519)	0.041
Heart failure decompensation	1.804 (1.180–2.157)	0.006	1.221 (0.762–1.957)	0.407	1.142 (0.716–1.820)	0.577	1.188 (0.743–1.900)	0.472	1.198 (0.755–1.899)	0.443
E/Ea	1.043 (1.013–1.074)	0.005					1.030 (0.998–1.063)	0.069		
SPAP	1.034 (1.021–1.047)	<0.001			1.013 (0.998–1.029)	0.530				
LVEF	0.936 (0.900–0.973)	0.001	0.9541 (0.914–0.996)	0.031						
TAPSE	0.979 (0.042–1.016)	0.266								
(IVCDi) × (S_T_/S_M_)	1.043 (1.030–1.057)	<0.001							1.027 (1.009–1.046)	0.003

Abbreviations: CKD; chronic kidney disease, NYHA; New York heart association, ACE-I/ARB: angiotensin-converting enzyme inhibitors/angiotensin receptor blocker, MRA: mineralocorticoid receptor antagonists, BNP; brain natriuretic peptide, E; early diastolic transmitral wave velocity, Ea; tissue Doppler mitral annular early diastolic velocity, LVEF; left ventricular ejection fraction, S_M_; mitral annulus peak systolic velocity, SPAB; systolic pulmonary artery pressure, S_T_; tricuspid annulus peak systolic velocity, TAPSE; tricuspid annular plane systolic excursion, IVCDi; inspiratory inferior vena cava diameter%.

**Table 6:** Cox proportional hazards models including all echo parameters to identify independent predictors of all-cause mortality.

Variables	HR 95%CI	*p*	HR 95%CI	*p*
Age (Years)	1.009 (1.030–1.052)	0.005		
Previous CKD	3.492 (2.284–5.341)	<0.001	2.100 (1.171–3.766)	0.013
NYHA class	3.314 (2.397–4.581)	<0.001	1.775 (1.234–2.554)	0.002
ACE-I/ARB usage	0.404 (0.266–0.615)	<0.001		
Loop diuretıcs usage	2.735 (1.452–5.152)	0.002		
MRA usage	0.664 (0.434–1.017)	0.060		
BNP	1.000 (1.000–1.000)	<0.001	1.000 (1.000–1.000)	<0.001
Appropriate shock	1.783 (1.005–3.160)	0.048	2.159 (1.174–3.974)	0.013
Heart failure decompensation	1.804 (1.180–2.157)	0.006		
E/Ea	1.043 (1.013–1.074)	0.005		
SPAP	1.034 (1.021–1.047)	<0.001		
LVEF	0.936 (0.900–0.973)	0.001	0.943 (0.902–0.985)	0.008
TAPSE	0.979 (0.042–1.016)	0.266		
(IVCDi) × (S_T_/S_M_)	1.043 (1.030–1.057)	<0.001	1.34 (1.016–1.053)	<0.001

**Table 7:** Cox proportional hazards models to identify independent predictors of MACE.

Univariate					Multivariate					
			Model 1		Model 2		Model 3		Model 4	
Variables	HR (95%CI)	*p*	HR (95%CI)	*p*	HR (95%CI)	*p*	HR (95%CI)	*p*	HR (95%CI)	*p*
Age (years)	1.019 (1.003–1.035)	0.019	0.996 (0.980–1.013)	0.654	0.991 (0.975–1.007)	0.269	0.991 (0.975–1.008)	0.298	0.9956 (0.978–1.011)	0.525
Male gender	1.842 (1.081–3.139)	0.025	1.634 (1.241–2.152)	<0.001	1.621 (1.230–2.135)	0.001	2.655 (1.531–4.604)	0.001	2.349 (1.346–4.099)	0.003
Previous CKD	3.947 (2.820–5.527)	<0.001	3.048 (1.999–4.647)	<0.001	2.508 (1.673–3.759)	<0.001	2.458 (1.641–3.681)	<0.001	2.556 (1.072–3.840)	< 0.001
NYHA	2.629 (2.081–3.320)	<0.001	2.223 (1.700–2.909)	<0.001	2.387 (1.821–3.129)	<0.001	2.381 (1.829–3.100)	<0.001	2.165 (1.647–2.846)	<0.001
ACE-I/ARB usage	0.412 (0.299–0.568)	<0.001	0.559 (0.395–0.791)	0.001	0.559 (0.393–0.795)	0.001	0.560 (0.395–0.795)	0.001	0.569 (0.403–0.806)	0.001
Loop diuretıcs usage	2.061 (1.340–3.168)	0.001	1.033 (0.644–1.655)	0.893	1.180 (0.738–1.887)	0.488	1.137 (0.714–1.811)	0.587	1.108 (0.692–1.774)	0.670
MRA usage	0.675 (0.487–0.935)	0.018	1.285 (0.872–1.892)	0.205	1.125 (0.779–1.627)	0.529	1.253 (0.856–1.834)	0.245	1.304 (0.883–1.926)	0.182
AF	1.459 (1.012–2.106)	0.043	1.128 (0.760–1.673)	0.551	1.215 (0.830–1.779)	0.403	1.161 (0.784–1.721)	0.456	1.083 (0.725–1.617)	0.237
CRT	0.601 (0.367–0.983)	0.043	0.769 (0.461–1.282)	0.314	0.774 (0.465–1.289)	0.325	0.716 (0.427–1.200)	0.205	0.734 (0.440–1.225)	0.237
BNP	1.000 (1.000–1.000)	<0.001	1.000 (1.000–1.000)	0.173	1.000 (1.000–1.000)	0.024	1.000 (1.000–1.000)	0.020	1.000 (1.000–1.000)	0.208
E/Ea	1.045 (1.021–1.070)	<0.001					1.033 (1.007–1.059)	0.011		
SPAP	1.032 (1.023–1.042)	<0.001			1.000 (0.987–1.013)	0.996				
LVEF	0.932 (0.905–0.961)	<0.001	0.942 (0.912–0.973)	<0.001						
TAPSE	0.997 (0.970–1.026)	0.857								
(IVCDi) × (S_T_/S_M_)	1.039 (1.029–1.050)	<0.001							1.018 (1.004–1.032)	0.013

Abbreviations: CKD; chronic kidney disease, NYHA; New York heart association, ACE-I/ARB: angiotensin-converting enzyme inhibitors/angiotensin receptor blocker, MRA: mineralocorticoid receptor antagonists, AF; atrial fibrillation, CRT; cardiac resynchronization therapy, BNP; brain natriuretic peptide, E; early diastolic transmitral wave velocity, Ea; tissue Doppler mitral annular early diastolic velocity, LVEF; left ventricular ejection fraction, S_M_; mitral annulus peak systolic velocity, SPAB; systolic pulmonary artery pressure, S_T_; tricuspid annulus peak systolic velocity, TAPSE; tricuspid annular plane systolic excursion, IVCDi; inspiratory inferior vena cava diameter.

**Table 8:** Cox proportional hazards models including all echo parameters to identify independent predictors of MACE.

Variables	HR (95%CI)	*p*	HR (95%CI)	*p*
Age (years)	1.019 (1.003–1.035)	0.019		
Male gender	1.842 (1.081–3.139)	0.025	2.491 (1.418–4.383)	0.002
Previous CKD	3.947 (2.820–5.527)	<0.001	3.111 (2.024–4.781)	<0.001
NYHA	2.629 (2.081–3.320)	<0.001	2.169 (1.648–2.854)	<0.001
ACE-I/ARB usage	0.412 (0.299–0.568)	<0.001	0.535 (0.377–0.760)	<0.001
Loop diuretıcs usage	2.061 (1.340–3.168)	0.001		
MRA usage	0.675 (0.487–0.935)	0.018		
AF	1.459 (1.012–2.106)	0.043		
CRT	0.601 (0.367–0.983)	0.043		
BNP	1.000 (1.000–1.000)	<0.001		
E/Ea	1.045 (1.021–1.070)	<0.001		
SPAP	1.032 (1.023–1.042)	<0.001		
LVEF	0.932 (0.905–0.961)	<0.001	0.947 (0.916–0.978)	0.001
TAPSE	0.997 (0.970–1.026)	0.857		
(IVCDi) × (S_T_/S_M_)	1.039 (1.029–1.050)	<0.001	1.017 (1.003–1.032)	0.021

Abbreviations: CKD; chronic kidney disease, NYHA; New York heart association, ACE-I/ARB: angiotensin-converting enzyme inhibitors/angiotensin receptor blocker, MRA: mineralocorticoid receptor antagonists, AF; atrial fibrillation, CRT; cardiac resynchronization therapy, BNP; brain natriuretic peptide, E; early diastolic transmitral wave velocity, Ea; tissue Doppler mitral annular early diastolic velocity, LVEF; left ventricular ejection fraction, S_M_; mitral annulus peak systolic velocity, SPAB; systolic pulmonary artery pressure, S_T_; tricuspid annulus peak systolic velocity, TAPSE; tricuspid annular plane systolic excursion. IVCDi; inspiratory inferior vena cava diameter.

## Data Availability

The data supporting this study’s findings are available from the corresponding author upon reasonable request.
